# A novel description of a syndrome consisting of *7q21.3* deletion including *DYNC1I1* with preserved *DLX5/6* without ectrodactyly: a case report

**DOI:** 10.1186/s13256-016-0921-8

**Published:** 2016-06-13

**Authors:** Héctor M. Ramos-Zaldívar, Daniel G. Martínez-Irías, Nelson A. Espinoza-Moreno, José S. Napky-Rajo, Tulio A. Bueso-Aguilar, Karla G. Reyes-Perdomo, Jimena A. Montes-Gambarelli, Isis M. Euceda, Aldo F. Ponce-Barahona, Carlos A. Gámez-Fernández, Wilberg A. Moncada-Arita, Victoria A. Palomo-Bermúdez, Julia E. Jiménez-Faraj, Amanda G. Hernández-Padilla, Denys A. Olivera, Kevin J. Robertson, Luis A. Leiva-Sanchez, Edwin Francisco Herrera-Paz

**Affiliations:** Campus San Pedro y San Pablo, School of Medicine, Universidad Católica de Honduras, San Pedro Sula, Honduras

**Keywords:** Deletion 7q21.3, Ectrodactyly, *SHFM1*, *DYNC1I1*, *DLX5/6*, DSS1, *COL1A2*, *SGCE*, *SLC25A13*

## Abstract

**Background:**

Chromosomal region *7q21.3* comprises approximately 5.2 mega base pairs that include genes *DLX5/6*, *SHFM1*, and *DYNC1I1* associated with split hand/split foot malformation 1. So far, there are reports of eight families with deletion of *DYNC1I1* and preserved *DLX5/6* associated with ectrodactyly. From these families, only three patients did not present ectrodactyly and, unlike our patient, no other cases have been described as having craniofacial dysmorphology, mitral valve prolapse, kyphoscoliosis, inguinal herniae, or personality disorder. There is no designation described in the literature for patients with syndromic manifestations without ectrodactyly, which hinders diagnosis.

**Case presentation:**

We report the case of a 44-year-old mestizo (combined European and Amerindian descent) man with a 3191 kilo base pairs deletion and International System for Human Cytogenetic Nomenclature array *7q21.3 (93,389,222-96,579,845)x1*. Clinical manifestations included micrognathia, retrognathia, wormian bones, auditory canal stenosis, depressed nasal bridge, epicanthal fold, fullness of upper eyelid, long philtrum, low-set ears, sensorineural hearing loss, kyphoscoliosis, bilateral inguinal herniae, mild mitral valve prolapse, and paranoid personality disorder. His isolated DNA was analyzed using a CytoScan HD Microarray system. Chromosome Analysis Suite software was utilized for the microarray analysis. All copy number changes were determined using the human genome build 19 (hg19/NCBI build 37).

**Conclusions:**

Cases of deletions within chromosome *7q21.3* that include the split hand/split foot malformation 1 region represent a diagnostic challenge when not presenting ectrodactyly despite being syndromic. Due to the heterogeneity of the region, a better method to group and classify these patients is needed to facilitate their clinical diagnosis. For this purpose, we suggest that patients with *7q21.3* deletion including *DYNC1I1* and preserved *DLX5/6* without ectrodactyly, accompanied by craniofacial dysmorphology, personality disorder, hearing loss, musculoskeletal disorder, inguinal herniae and/or mitral valve prolapse be referred to by the eponym Ramos–Martínez syndrome.

**Electronic supplementary material:**

The online version of this article (doi:10.1186/s13256-016-0921-8) contains supplementary material, which is available to authorized users.

## Background

Chromosome 7 encompasses approximately 158 mega base pairs (Mb) and 1917 genetic structures [1]. More than 360 diseases are associated to genes that reside within this chromosome [1]. The identification of the genes for cystic fibrosis (cystic fibrosis transmembrane conductance regulator, *CFTR*; Mendelian Inheritance in Man, MIM 602421) and erythropoietin (*EPO*; MIM 133170), as well as gene families such as the T-cell receptor gene family and the homeobox A gene family have made chromosome 7 a focus of interest for human genetics research.

Within this chromosome is region *7q21.3* which comprises approximately 5.2 Mb [2]. Among the most important disorders found in this region are Ehlers–Danlos syndrome type VIIB (MIM 130060), osteogenesis imperfecta types II to IV (MIM 166210, 259420, 166220), neonatal-onset type II citrullinemia (MIM 605814), and split hand/split foot malformation 1 (SHFM1; MIM 183600). Aberrations in this band are characterized by wide heterogeneity and variable expression that represent a challenge for clinical diagnosis.

The genes within the SHFM1 region participate in proper limb development. These include distal-less homeobox 5 (*DLX5*; MIM 600028)*,* distal-less homeobox 6 *(DLX6*; MIM 600030), deleted in split hand/split foot malformation 1 region (*SHFM1*; MIM 601285), and dynein cytoplasmic 1 intermediate chain 1 (*DYNC1I1*; MIM 603772) through its *exonic enhancers* (*eExons*) *15* and *17*. So far, there are reports of eight families with deletion of *DYNC1I1* and preserved *DLX5/6* associated with ectrodactyly, the main manifestation of the SHFM1 syndrome [3]. We report a case that involves a deletion of 3 Mb in chromosome *7q21.3* region including *DYNC1I1* with preserved *DLX5/6* without ectrodactyly accompanied by craniofacial dysmorphology, personality disorder, hearing loss, musculoskeletal manifestations, bilateral inguinal herniae, and mitral valve prolapse.

## Case presentation

We report the case of a 44-year-old mestizo (combined European and Amerindian descent) man born to non-consanguineous parents. He is the first of two children conceived by a 25-year-old mother and a 34-year-old father; he has no relevant prenatal history and no known family history of congenital malformations, although his father was diagnosed with schizophrenia. There was no known exposure to teratogenic drugs, infections, or radiation. He was born at 41-week gestation by normal vaginal delivery.

He underwent multiple reconstructive surgeries 15 years prior to our evaluation, where mandibular hypoplasia and prominent low-set ears were corrected. Photographs from childhood showed epicanthal fold, fullness of upper eyelid, depressed nasal bridge, anteverted nares, long philtrum, malocclusion, micrognathia, retrognathia, full cheeks, and prominent low-set ears.

His first physical evaluation at our institution was performed at age 42. Clinical findings included male pattern baldness and black hair with multiple strands of gray hair, stenosis of the cartilaginous portion of his external auditory canal and low-set ears, long uvula, enlarged thyroid gland, mitral murmur, kyphoscoliosis, bilateral inguinal herniae, erythematous and scaly lesions of his feet consistent with tinea pedis, as well as discolored yellow-green nails consistent with onychomycosis.

**Fig. 1:**
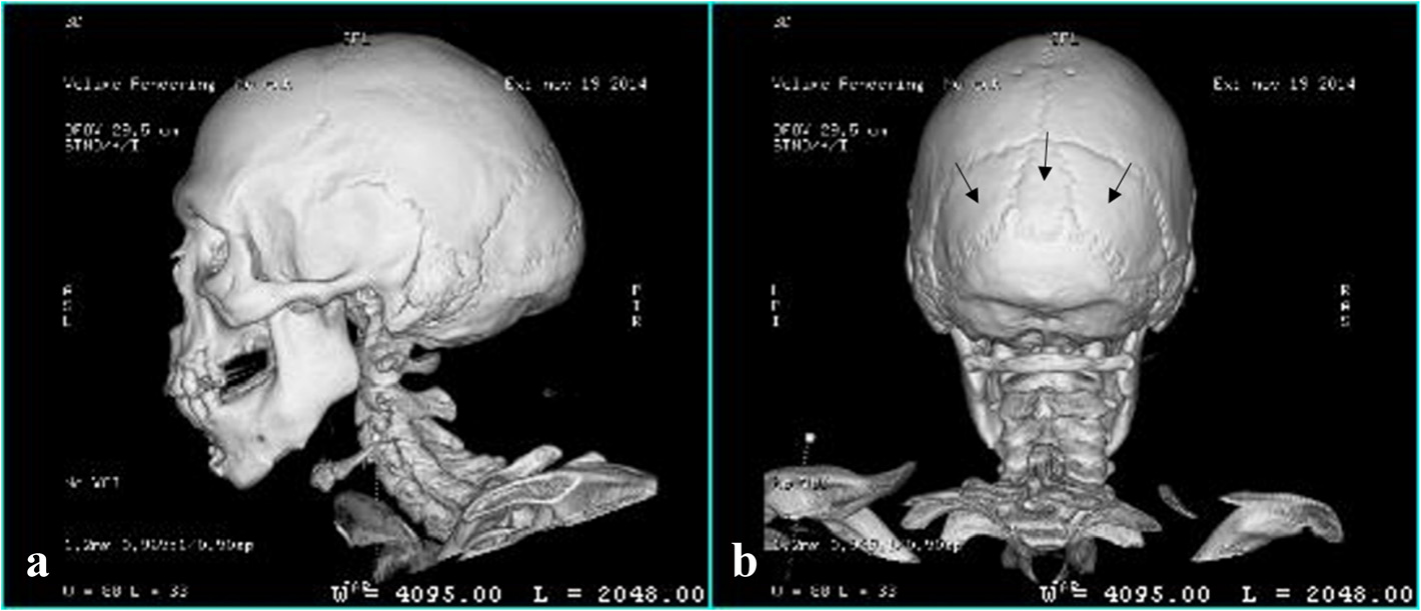
Three-dimensional reconstruction of the computed tomography of the patient’s head. **a** Lateral view shows surgical evidence in inferior maxillary bone, showing signs of microgenia, retrognathia, and a slight abnormality of dental occlusion. **b** Posterior view shows wormian bones in his occipital region

Echocardiography revealed impaired relaxation grade 1 and mild prolapse of the anterior leaflet of his mitral valve. Audiometry reported bilateral sensorineural hearing loss; the test was limited by the presence of cerumen impaction mainly in his right auditory canal. Computed tomography revealed surgical evidence in inferior maxillary bone, showing signs of microgenia, retrognathia, and a slight abnormality of dental occlusion. His infratentorial and supratentorial brain parenchyma was of normal density. Wormian bones were identified in his occipital cranial region (Fig. 1). The shape of his skull had a mild dolichocephalic configuration. His right ear presented cerumen impaction of 16.0×10.5 mm. His middle and inner ear structures were without abnormalities.

A Minnesota Multiphasic Personality Inventory-2 (MMPI-2) [4] showed an elevated psychological profile on the scales of hysteria, hypochondriasis, and paranoia; the patient appeared to be socially balanced, extraverted, and open to new experiences, as well as spontaneous, controlling, excessively rational, rigid, egocentric, defensively paranoid, power-oriented, interiorly distrustful, suspicious, hostile, and extremely avoidant of criticism. In general, he was very calm, but prone to periods of anxiety, tension, and somatic symptoms. The results of a House-Tree-Person [5] test concurred with the previously described findings. Based on the evaluations, a paranoid personality disorder was established. An intellectual quotient of 90, a verbal intellectual quotient of 92, and a performance intellectual quotient of 84 were reported.

Additional laboratory analyses showed: thyroid function test results of total and free triiodothyronine (T_3_), total and free thyroxine (T_4_), and thyroid-stimulating hormone (TSH) to be within normal ranges; a slight increase in total cholesterol levels with a value of 222 mg/dl; high-density lipoprotein (HDL) of 43 mg/dl and low-density lipoprotein (LDL) of 128 mg/dl; elevated triglycerides with a value of 234 mg/dl; no electrolyte abnormalities. His levels of glycemia, creatinine, transaminases, bilirubin, and testosterone were within normal ranges.

A total of 15 cells in metaphase were analyzed through conventional G-band karyotyping, reporting normal 46,XY. Due to the patient’s language characteristics as perceived by the examiner (verbiage), the affinity for music showed by the patient (multi-instrumentalist), complemented with the craniofacial dysmorphology, Williams–Beuren syndrome (MIM 194050) was suspected. A score of 8 was obtained when the Williams syndrome scoring table, which was presented at the 1994 Williams Syndrome Association Convention in San Diego, California, was applied; if the score ≥3, fluorescence *in situ* hybridization (FISH) studies should be considered [6].

A 7 ml whole blood sample was extracted for analysis. To rule out aberrations related to the Williams–Beuren syndrome, a FISH was performed using a probe specific for the elastin gene (*ELN*; MIM 130160) in the Williams syndrome critical region on chromosome 7. In an individual who does not have the deletion, four signals will be detected, two signals on each chromosome 7. The signal at *7q11.23* is specific for the elastin gene, while the signal at *7q36* is the *D7S427* chromosome 7 control probe which facilitates identification of the chromosome 7 homologs. In this study, 25 of 25 metaphase spreads and 65 of 66 interphase nuclei examined produced four signals indicating that there was no deletion in this region [see Additional file 1].

FISH analysis does not cover all genes involved in Williams–Beuren syndrome within region *7q11.23*. Therefore, a whole genome single nucleotide polymorphism (SNP) array was performed. A second blood sample was extracted and DNA was analyzed using a CytoScan HD Microarray system (Affymetrix). This platform consists of 2.67 million markers (comprising ~1.9 million non-polymorphic copy number probes and ~750,000 SNP probes) at an average spacing of 1 probe every 800 base pairs (bp) throughout the entire human genome. This test compares the patient’s sample with control samples from the HapMap set of 270 individuals, and identifies genomic copy number variations and loss of heterozygosity regions. Chromosome Analysis Suite (ChAS) was utilized for the analysis of this microarray. SNP genotyping on this platform has the enhanced ability to identify long contiguous stretches of homozygosity (LCSH) and uniparental disomy; however, this assay cannot detect polyploidy, balanced rearrangements (for example, inversions and balanced chromosomal translocations), point mutations, and most mosaic conditions. All copy number changes were determined using the human genome build 19 (hg19/NCBI build 37).

Microarray CytoScan revealed 3191 kilo base pairs (kb) in a different region of chromosome 7 with International System for Human Cytogenetic Nomenclature (ISCN) array (hg19) *7q21.3 (93,389,222-96,579,845)x1*. This deletion included SHFM1 syndrome region and 31 genes, of which 17 have Online Mendelian Inheritance in Man (OMIM) entries. The OMIM genes within this region are *TFPI2* (MIM 600033), *GNGT1* (MIM 189970), *GNG11* (MIM 604390), *BET1* (MIM 605456), collagen type 1 alpha-2 (*COL1A2*; MIM 120160), *CASD1* (MIM 611686), sarcoglycan epsilon (*SGCE*; MIM 604149), *PEG10* (MIM 609810), *PPP1R9A* (MIM 602468)*, PON1* (MIM 168820), *PON3* (MIM 602720), *PON2* (MIM 602447), *ASB4* (MIM 605761), *PDK4* (MIM 602527), *DYNC1I1*, solute carrier family 25 (citrin) member 13 (*SLC25A13*; MIM 603859), and *SHFM1*. The deletion did not include genes *DLX5/6* [see Additional file 2].

Five maternal family members who showed mild mandibular hypoplasia and epicanthal fold were also analyzed. Microarray CytoScan did not report abnormalities in copy number of known or potential significance for the regions included on the chip. No cytogenetic analyses were performed on the patrilineal side of the patient. The father died before the patient was identified, and there were no available family members from the patrilineal side for genetic study.

## Discussion

We described a patient with craniofacial dysmorphology and paranoid personality disorder with a 3 Mb deletion in chromosome *7q21.3*. In this case, a deletion of *DYNC1I1* gene with preserved *DLX5/6* without ectrodactyly was reported. In addition, he presented musculoskeletal disorder, bilateral inguinal herniae, mitral prolapse, and auditory canal stenosis.

There are reports of eight families with deletion of *DYNC1I1* and preserved *DLX5/6* associated with ectrodactyly, but with decreased penetrance [3]. Delgado and Velinov reported the case of a family (the father and three of his sons) with a 1 Mb deletion associated to various degrees of intellectual disability; only two of the male siblings had ectrodactyly [3]. However, echocardiography was normal in all cases and no craniofacial dysmorphology was described. Tayebi *et al*. described four families with deletions of 167 kb (family 1), 510 kb (family 2), 205 kb (family 3), and 169 kb (family 4) [7]. Families 1, 3, and 4 presented SHFM1 without hearing loss. Family 2 presented SHFM1 with hearing loss. Unlike our case, none of the families reported craniofacial dysmorphology. Mitral valve prolapse, personality disorder, neurological or motor abnormalities were not reported. Rattanasopha *et al*. reported the case of a family of 10 members with a 103 kb deletion, of which eight presented SHFM1 with variable severity and two did not present ectrodactyly [8]. Unlike our patient, none of the cases reported ear abnormalities. The two family members described by Rattanasopha without SHFM1 did not present any other associated abnormality. Lango Allen *et al*. reported the case of a family of two members with a 106 kb deletion with ectrodactyly, in which hearing loss was the only associated manifestation described [9]. Kouwenhoven *et al*. described a patient with 880 kb deletion with non-syndromic SHFM1 [10]. Genomic locations and phenotypes for the nine families, including the present case report, are shown in Table 1.

**Table 1:** Genomic locations and phenotypes of nine families associated with deletion of *DYNC1I1* and preserved *DLX5/6*

Family (authors and Reference number)	Genomic location (hg19)	Size of deletion (kb)	Phenotype	
This case report	chr7: 93,389,222-96,579,845	3191	Craniofacial dysmorphology, personality disorder, hearing loss, musculoskeletal disorder, inguinal herniae, mitral valve prolapse, without ectrodactyly	
Tayebi *et al*. [7] family 1	chr7:95,615,187-95,783,313	167	SHFM	
Tayebi *et al*. [7] family 2	chr7:95,624,825-96,135,521	510	SHFM, hearing loss	
Tayebi *et al*. [7] family 3	chr7: 95,667,046-95,872,044	205	SHFM	
Tayebi *et al*. [7] family 4	chr7:95,693,341-95,862,369	169	SHFM	
Delgado and Velinov [3]	chr7:94,769,383-95,801,045	1000	Two members	Intellectual disability, SHFM
			Two members	Intellectual disability, without SHFM
Rattanasopha *et al*. [8]	N/A	103	Eight members	SHFM
			Two members	No abnormalities
Lango Allen *et al*. [9]	N/A	106	SHFM, hearing loss	
Kouwenhoven *et al*. [10]	N/A	880	Non-syndromic SHFM	

*N/A* not available, *SHFM* split hand/split foot malformation

The deletion of *COL1A2* gene might be the primary cause for the development of mitral valve prolapse identified in our patient. However, as described by Hirschfeld *et al*., abnormalities of the thoracic skeleton, such as scoliosis, are associated with the development of secondary mitral prolapse [11]. Mutations of the *COL1A2* gene have been related to bilateral inguinal herniae and wormian bones in Ehlers–Danlos syndrome type VIIB [12, 13].

The deletion of *SGCE* gene has been related with psychosis and cognitive impairment, and might be responsible for the paranoid personality disorder of our patient. Dale *et al*. reported the case of a family with a 0.17 Mb deletion that involved 90 % of *SGCE*, describing it as a risk factor for psychiatric diseases [14]. It is noteworthy that the father of the index case in this report was diagnosed with paranoid schizophrenia, with a high incidence of psychosis in the extended family. Genetic analyses of the patrilineal side of our patient would have been convenient because his father was diagnosed with schizophrenia.

*DYNC1I1* gene contains *eExons 15 and 17*, which interact with *DLX5* and *DLX6* in limb development [15]. Aberrations that involve these enhancers have been associated with SHFM1, hearing loss, craniofacial malformations, and intellectual disability [15, 16]. Our patient did not present ectrodactyly, even though this gene was included in the deletion. This could be explained by the incomplete penetrance described in this region [3]. In addition, Hong *et al*. described the existence of secondary enhancers (shadow enhancers) that explain why deletions of well-defined enhancers sometimes do not produce expected phenotypes, a mechanism that could account for the normal limb development of our patient [17].

*SLC25A13* contains the intragenic enhancer *hs1642*, which has been associated with hearing loss [7]. The deletion of this gene could explain the sensorineural hearing loss reported in the audiometry of our patient. However, the auditory canal stenosis and the tendency to accumulate cerumen are other factors that contribute to the hearing loss of our patient.

The *SHFM1* gene has been associated with multiple craniofacial malformations, such as micrognathia, retrognathia, auditory canal stenosis, low-set ears, and depressed nasal bridge [16, 18–20]. The craniofacial dysmorphology of our patient could be explained by the deletion of this gene.

## Conclusions

Among the families reported so far with *DYNC1I1* deletion and preserved *DLX5/6*, the 3191 kb deletion of our patient is the most extensive. From the other eight families, only three patients did not present ectrodactyly and no cases described craniofacial dysmorphology, mitral valve prolapse, kyphoscoliosis, inguinal herniae, or personality disorder. These phenotypical differences might be caused by the deletion of more genes in our patient.

Cases of deletions within chromosome *7q21.3* that include the SHFM1 region represent a diagnostic challenge when not presenting with ectrodactyly, despite being syndromic. Due to the heterogeneity of the region, a better method to group and classify these patients is needed to facilitate their clinical diagnosis. For this purpose, we suggest that patients with *7q21.3* deletion including *DYNC1I1* and preserved *DLX5/6* without ectrodactyly, accompanied by craniofacial dysmorphology, personality disorder, hearing loss, musculoskeletal disorder, inguinal herniae and/or mitral valve prolapse be referred to by the eponym Ramos–Martínez syndrome.

## Patient’s perspective

This is an encouraging moment; a special Sunday when I face the possibility of accomplishing something I longed for all my life. As I listen to Chopin’s preludes, I feel immensely grateful because life has given me this experience; God has been very loving and caring with me. This gratitude extends to this prestigious journal, the *Journal of Medical Case Reports*, which gives me the blessed opportunity to express my perspective as a patient. In my view, this is very admirable since years ago journals only published the medical report without regard for what the patient felt or wanted to say. Whatever the reason for the decision made by the journal, I thank you deeply for taking the time to read these simple lines. This only confirms my admiration for all your excellent work and distinguished social projection in such a complicated, but no less interesting, area as is medicine with all of its wonderful and useful contributions to humanity.

Ever since I was born, the love of my family made them strive to provide me with the best conditions according to their means. They did so by placing me in the hands of the best doctors of my country, Honduras, in Central America. So without knowing exactly what was happening, and almost by inertia, they sought the help of many different specialists: pediatricians, otolaryngologists, and others. Unfortunately, they could never give me a definitive diagnosis. That taught me the discipline of taking care of my own health. Later, from the age of 17 onwards, I personally searched for professional help due to the discomforts I felt; more specifically, I consulted a plastic surgeon, a cardiologist, a dermatologist, a gastroenterologist, an orthopedist, and general practitioners. With your permission, I will use an analogy to explain the symptoms I have experienced:

Long ago Copernicus made public the reality, now proven by science, that our planet Earth revolved around the Sun and not the other way around. Many things that were not previously accepted have been uncovered by science and we are still shrouded in the astonishing mystery of life since the Big Bang started to expand our universe. I have noticed in time that the world does not revolve around my existence, but it is my being that depends on the laws of life (just as I am). What am I? Nothing more than one of the countless forms of life that constitutes part of this vast and deep universe, like some kind of nanocosmos (DNA). Some time passed before I began to suspect the true nature of my genetics. I was born almost like everyone else, but without knowing there was a small flaw in my genetics. God using those angelical beings, as I like to call doctors and nurses, was helping me to realize what was happening to me. It was evident that I was born with low-set ears, a small chin, and other features would become more noticeable in time. The love of my blessed parents and my phenomenal family raised me in an environment full of joy, of music, of reunion, of prayer, and of fraternity. Amid all these symptoms, using the analogy above, there was still an interesting and sensible path to travel.

I noticed that the visits to the doctors became more frequent and sensed that beyond a general medical examination something was going on, but I could not explain it. At home, I was always treated as a normal person, a child like everyone else. It was not until the fifth year in school that I began to be a victim of bullying. This was like a kind of tunnel in my life, the years went by, and I even lost three years of high school because I did not know how to handle these emotions. When I started my adolescence, I noticed that my open bite was more apparent, even the shape of my skull bulging from behind, excessive acne that once required emergency surgery, and suffering from four fissures in my feet, stomach aches, anxiety, frequent headaches, and a level of depression that only believing in God and feeling loved by my family and my friends gave me the hope and strength to carry on.

It was not until several years later that, realizing that there was a research team at the *Universidad Católica de Honduras*, I sought professional help that was immediately and gladly accepted, leading to a series of general and specific tests. On the one hand I felt normal because I had already been through hospitals and other medical centers; on the other hand, I felt very optimistic because it meant the possibility of really discovering who I was genetically. I did not suffer any discomfort during these tests, just a feeling of anxiety while staying still during tomography, and some weakness during the extraction of blood samples. However, I felt that God was with me more and more because we were working with the scientific method to help me elucidate my case.

At my 44 years of age I still feel like an adolescent and want to experience life to its fullest. I try to enjoy everything that happens to me. I still periodically feel low blood pressure and deafness. I get tired more easily, with pain when I walk a lot and excel myself due to some inguinal hernias. I see flies or black spots, and I also feel anxiety, but with a little more self-control. However, these are small things compared to the improvement I have felt knowing the result of my genetic study. Now I see a little more clearly and know myself much better. I love to always enjoy my family, my mother, my sister, my little nieces and nephew, and others, including my pets, my time alone by myself, my friends, my work environment, and anywhere I go. I have a vital strength; I like to walk a lot, observe life, and think that even though I am not much different from what I was before starting the genetic test now I have a greater awareness of my life. There are endless things that I would like to share and will finish with this poem:

I will never be able to explain it humanly: God exists; my chromosome 7 demonstrates it; rain of pure love; mysterious DNA; missing link whose mercy finds and restores; I have wandered like the people of Israel for 44 years and the uncreated light; immortal God; reveals His mercy in the unusual. Like a gipsy I went from doctor to doctor; from hospital to operating room; he shall not live; was dictum of science when they saw my birth; but God decided otherwise; before seeing Promised Land; he shall overcome great challenges; then he will know who he is; and there came His embrace of light during my grief; I suffered without knowing that in pain God peered, hidden and suspicious, for his plan was unforeseen; He concealed 17 genes that would shout with time; all his heart will sigh life; and I survived; and He gave me the musical angel; I will give him my grace and he will live. And I was invited to the school of pain; only that with every blow his love was more abundant; and I sang to the rays of the silver moon; and I wrote to the deserts where He took me; and He put before me an oasis of peace and reflection; and I acted with the script of my own story; everything and everyone has Christ sent to call me to the permanent celebration inside my soul; musicalizes with notes of Andromeda’s perfume; even what I do not understand; everything is a blessing; and as the people of Israel had their Messiah; that gentiles at their compass would receive; in the same way I received that good news; God knows what is best; like everyone I have human flaws and miseries, maybe more than others; but well above my clay and molecules, I came to tell the world that God is love and that it is possible to be happy; even within my syndrome; happiness is you; because God lives within you; so many things I would want to write; but my clumsy inspiration leaves me with nothing more; it is so great the desire of saying a new language; the one God made when creating the Big Bang; that I would never end even at a rate of light speed to the infinite power. Oh heart that has received countless emotions; go ahead without faltering; more than trigonometric formulas, laboratories, and genetics; I put in the hands of God and science, what is left to live, in harmony.

## Additional files

Additional file 1: Fluorescence *in situ* hybridization. Fluorescence *in situ* hybridization was performed using a probe specific for the elastin gene (*ELN*) in the Williams syndrome critical region on chromosome 7. (PDF 12 kb)

Additional file 2: Microarray CytoScan. DNA analysis using a CytoScan HD Microarray system (Affymetrix). (PDF 20 kb)

## Abbreviations

*COL1A2*: collagen type 1 alpha-2
*DLX5*: distal-less homeobox 5
*DLX6*: distal-less homeobox 6
*DYNC1I1*: dynein cytoplasmic 1 intermediate chain 1
*eExons*: *exonic enhancers*
FISH: fluorescence *in situ* hybridization
kb: kilo base pairs
Mb: mega base pairs
MIM: Mendelian Inheritance in Man
MMPI-2: Minnesota Multiphasic Personality Inventory-2
OMIM: Online Mendelian Inheritance in Man
*SGCE*: sarcoglycan epsilon
*SHFM1*: deleted in split hand/split foot malformation 1 region
SHFM1: split hand/split foot malformation 1
*SLC25A13*: solute carrier family 25 (citrin) member 13
SNP: single nucleotide polymorphism
