# Supplementary material for: A novel description of a syndrome consisting of 7q21.3 deletion including DYNC1I1 with preserved DLX5/6 without ectrodactyly: a case report
Source: J Med Case Rep. 2016 Jun 13;10:156. doi: 10.1186/s13256-016-0921-8 (PMC4904365; doi:10.1186/s13256-016-0921-8)
Supplement: Additional file 1: — Fluorescence in situ hybridization. Fluorescence in situ hybridization was performed using a probe specific for the elastin gene (ELN) in the Williams syndrome critical region on chromosome 7. (PDF 12 kb) [file 13256_2016_921_MOESM1_ESM.pdf]

Greenwood Genetic Center

CYTOGENETIC LABORATORY

125 Gregor Mendel Circle
Greenwood, SC 29646
1-800-473-9411 FAX
864-941-8133

Patient Sample

Study #: **14-04296FWMS**

Date of birth: **4/30/1971**

Type of Specimen: **Peripheral blood**

Requested by: **Edwin Francisco Herrera Paz, M.D.**

Test Requested: **Fluorescence in situ hybridization**

Reason for Request: **Rule out Williams syndrome, Male with distinctive facial features during infancy, Broad forehead, Medial eyebrow flare, Periorbital**

Collection Date: **3/12/2014**

Referral ID#: **N/A 0501 1971 02759**

Sample Received: **3/13/2014**

Test Request Received: **3/13/2014**

Date Reported: **3/19/2014**

Results & Interpretation

ISCN Nomenclature: ish 7q11.23(ELNx2)

No evidence of a microdeletion in the Williams syndrome critical region was detected by FISH.

Comment: Fluorescence in situ hybridization (FISH) was performed using a probe specific for the elastin gene [ELN] in the Williams Syndrome Critical Region on chromosome 7 (Vysis #32-190041). In an individual that does not have the deletion, four signals will be detected- two signals on each chromosome 7. The signal at 7q11.23 is specific for the elastin gene, while the signal at 7q36 is the D7S427 Chromosome 7 control probe which facilitates identification of the chromosome 7 homologs.

In the present study, 25 of 25 metaphase spreads and 65 of 66 interphase nuclei examined produced four signals indicating that this region is not deleted.

Reviewed and electronically signed by
Barbara R. DuPont, PhD, FACMG
Senior Director, Cytogenetics Laboratory
cc: Hector Miguel Ramos Zaldivar, M.S.

This test was developed and its performance determined by the Greenwood Genetic Center. It is used for clinical purposes. It has not been cleared or approved by the U.S. Food & Drug Administration. The FDA has determined that such clearance or approval is not necessary. This laboratory is certified under the Clinical Laboratory Improvement Amendments of 1988 as qualified to perform high complexity clinical testing.
