# Supplementary material for: A novel description of a syndrome consisting of 7q21.3 deletion including DYNC1I1 with preserved DLX5/6 without ectrodactyly: a case report
Source: J Med Case Rep. 2016 Jun 13;10:156. doi: 10.1186/s13256-016-0921-8 (PMC4904365; doi:10.1186/s13256-016-0921-8)
Supplement: Additional file 2: — Microarray CytoScan. DNA analysis using a CytoScan HD Microarray system (Affymetrix). (PDF 20 kb) [file 13256_2016_921_MOESM2_ESM.pdf]

Greenwood Genetic Center

106 Gregor Mendel Circle
Greenwood, SC 29646
PHONE:1-800-473-9411
FAX: 864-941-8133
www.ggc.org

CYTOGENETIC LABORATORY

Patient Sample

Study #: **14-15938CMCS**

Date of birth:

Type of Specimen: **DNA from blood**

Requested by: **Edwin Francisco Herrera Paz, M.D.**

Test Requested: **Microarray CytoScan**

Reason for Request: **Distinctive Facial features during Infancy, Broad Forehead, Medial Eyebrow Flare**

Collection Date: **10/8/2014**

Referral ID#: **0501197102759**

Sample Received: **10/9/2014**

Test Request Received: **10/9/2014**

Date Reported: **10/30/2014**

Results & Interpretation

ISCN Nomenclature: arr 7q21.3(93,389,222-96,579,845)x1

Results:

#	Chr	Cytoband	Start	Stop	Size	Gain/Loss	Interpretation	Inheritance
1	7	q21.3	93,389,222	96,579,845	3191kb	loss	Pathogenic	

Interpretation:

CNV 1: This region includes the Split hand/foot malformation 1 (SHFM1) syndrome region and includes 31 genes, of which 17 have OMIM entries. The OMIM genes within this region are *TFPI2, GNGT1, GNG11, BET1, COL1A2, CASD1, SGCE, PEG10, PPP1R9A, PON1, PON3, PON2, ASB4, PDK4, DYNC1I1, SLC25A13, SHFM1*. SHFM1 is an autosomal dominant disorder with reduced penetrance and variable expressivity.
A detailed account of some of the genes within this deleted region are as follows:
Mutations in the *COL1A2* gene are associated with osteogenesis imperfecta types I-IV, Ehlers-Danlos syndrome type VIIB, recessive Ehlers-Danlos syndrome Classical type, idiopathic osteoporosis, and atypical Marfan syndrome [provided by R. Dalgleish, Feb 2008]. Mutations in *SGCE* are associated with myoclonusdystonia and epilepsy (*J Neurol.* 2014 Feb;261(2):358-62). Mutation in the *SLC25A13* gene causes neonatalonset type II citrullinemia, also known as neonatal intrahepatic cholestasis caused by citrin deficiency (NICCD). NICCD is an autosomal recessive metabolic disorder characterized by poor growth, intrahepatic cholestasis, and increased serum citrulline (*Int. J. Molec. Med.* 28: 33-40, 2011; *J. Hepatol*. 49: 810-820, 2008). The *SHFM1* gene has been proposed to be a candidate gene for the autosomal dominant form of the heterogeneous limb developmental disorder split hand/split foot malformation type 1. Splithand/split-foot malformation (SHFM) is a limb malformation involving the central rays of the autopod and presenting with syndactyly, median clefts of the hands and feet, and aplasia and/or hypoplasia of the phalanges, metacarpals, and metatarsals. Some patients with SHFM1 have been found to have intellectual disability, ectodermal and craniofacial findings, orofacial clefting (*Am J Med Genet A.* 2006 Jul 1;140(13):1419-27). Some cases of splithand/foot malformation-1 (SHFM1) represent a contiguous gene syndrome caused by deletion, duplication, or rearrangement of chromosome 7q21.3 involving the *DSS1*, *DLX5*, and *DLX6* genes. However, *DSS1*, *DLX5*, and *DLX6* are not included in this deletion.

Parental studies are recommended to determine whether this copy number alteration is inherited or *de novo.* If requested, parental studies can be performed for a fee. All other copy number changes were smaller than 0.2

Mb or were in regions of known copy number variants. To ensure the best medical care possible, genetic counseling is recommended.

Patient Sample

Study #: **14-15938CMCS**

Date of birth: **4/30/1971**

Type of Specimen: **DNA from blood**

Comment:

The isolated DNA was analyzed using the Affymetrix CytoScan HD Microarray system. This platform consists of 2.67 million markers (comprised of ~1.9 million non-polymorphic copy number and ~750,000 single nucleotide polymorphism (SNP) probes) at an average spacing of 1 probe every 800 bp throughout the entire human genome. This test compares the patient sample to control samples from the HapMap set of 270 individuals. The Affymetrix CytoScan HD microarray is a diagnostic assay used by the Greenwood Genetic Center for the identification of genomic copy number variations and loss of heterozygosity regions. Chromosome Analysis Suite (ChAS) software has been utilized for the analysis of this microarray. SNP genotyping on this platform has the enhanced ability to identify long contiguous stretches of homozygosity (LCSH) and uniparental disomy; however, this assay cannot detect polyploidy, balanced rearrangements (eg. inversions and balanced chromosomal translocations), point mutations, and most mosaic conditions. All copy number changes were determined using the human genome build 19 (hg19/NCBI build 37).

Reviewed and electronically signed by
Alka Chaubey, PhD, FACMG
Director, Cytogenetics Laboratory cc:
Hector Miguel Ramos Zaldivar, M.S.

This test was developed and its performance determined by the Greenwood Genetic Center. It is used for clinical purposes. It has not been cleared or approved by the U.S. Food & Drug Administration. The FDA has determined that such clearance or approval is not

necessary. This laboratory is certified under the Clinical Laboratory Improvement Amendments of 1988 as qualified to perform high complexity clinical testing.
